# Glycaemic Control and Associated Self-Management Behaviours in Diabetic Outpatients: A Hospital Based Observation Study in Lusaka, Zambia

**DOI:** 10.1155/2016/7934654

**Published:** 2015-12-21

**Authors:** Emmanuel Mwila Musenge, Charles Michelo, Boyd Mudenda, Alexey Manankov

**Affiliations:** ^1^Department of Physiological Sciences, School of Medicine, University of Zambia, Ridgeway Campus, P.O. Box 50110, 10101 Lusaka, Zambia; ^2^Department of Public Health, Section for Epidemiology and Biostatistics, School of Medicine, University of Zambia, Ridgeway Campus, P.O. Box 50110, 10101 Lusaka, Zambia

## Abstract

*Background*. The control of *diabetes mellitus* depends on several factors that also include individual lifestyles. We assessed glycaemic control status and self-management behaviours that may influence glycaemic control among diabetic outpatients. *Methods*. This cross-sectional study among 198 consenting randomly selected patients was conducted at the University Teaching Hospital diabetic clinic between September and December 2013 in Lusaka, Zambia. A structured interview schedule was used to collect data on demographic characteristics, self-management behaviours, and laboratory measurements. Binary logistic regression analysis using IBM SPSS for Windows version 20.0 was carried out to predict behaviours that were associated with glycaemic control status. *Results*. The proportion of patients that had good glycaemic control status (HbA_1c_≤ 48 mmol/mol) was 38.7% compared to 61.3% that had poor glycaemic control status (HbA_1c_≥ 49 mmol/mol). Adherence to antidiabetic treatment and fasting plasma glucose predicted glycaemic control status of the patients. However, self-blood glucose monitoring, self-blood glucose monitoring means and exercise did not predict glycaemic control status of the patients.  *Conclusion*. We find evidence of poor glycaemic control status among most diabetic patients suggesting that health promotion messages need to take into account both individual and community factors to promote behaviours likely to reduce nonadherence.

## 1. Introduction

*Diabetes mellitus* (DM) is a group of metabolic diseases of prolonged hyperglycaemia due to either the pancreas not producing enough insulin, or the cells of the body not responding properly to the insulin produced [1]. It is a major public health problem that is approaching epidemic proportions worldwide [2] and largely associated with lifestyle changes in emerging economies, a double edged sword [3]. The worldwide prevalence of both types 1 and 2 DM among adults was 285 million (6.4%) in 2010 and is predicted to rise to around 439 million (7.8%) by 2030 [4]. Although sub-Saharan Africa has been reported to have an estimated DM adult prevalence of 2.4%, this is probably not just an understatement but the burden is also likely to increase in a few years' time [5].

The progressive nature of the disease requires regular monitoring of glycaemia and, when necessary, intensification of any existing treatment [6]. The most common form of monitoring involves pricking the fingertip to obtain a blood sample, which is tested with a glucometer to determine the patient's blood glucose level. The results from self-glucose monitoring aid the diabetics in decision making on the food, exercise, and use of medications including dose adjustment. This allows diabetics to manage their disease and avoid associated complications of uncontrolled abnormal plasma glucose levels. For type 2 DM, self-management behaviours are an important aspect of management and should be recommended for all diabetic patients. These self-management behaviours include, but are not limited to, adherence and self-blood glucose monitoring (SBGM) as well as exercise and body mass index (BMI) monitoring [7].

One way of monitoring glycaemia reliably is by measuring glycated haemoglobin (HbA_1c_). Glycated haemoglobin is determined by colorimetry and turbidimetry using the clinical chemistry analyzer. High level of glycated hemoglobin indicates poor control of diabetes and is associated with cardiovascular disease, nephropathy, and retinopathy [8]. Glycated haemoglobin is the primary target of glycaemic control. Measuring HbA_1c_ can be used to reflect average blood glucose levels over the previous 8 to 12 weeks prior to the measurement, thus providing a useful longer-term gauge of glycaemic control [9]. The HbA_1c_ test should be done approximately every 3 months in uncontrolled or at least twice a year in well-controlled diabetic patients.

A number of studies have been conducted among diabetic patients in different parts of the world and most of them aimed to estimate the exact burden and associated factors. In a study by Mahmood and Aamir in Pakistan, over half of the patients had poor diabetic control [10]. Moreira Jr. et al. [11] in Venezuela reported 87% poor glycaemic control status in type 1 and 75% in type 2 diabetic patients. Sobngwi et al. [12] in a six sub-Saharan African countries study revealed that only 29% of the patients had good glycaemic control. The background retinopathy (18%) and cataract (14%) were the most common eye complications while macrovascular disease was rare, and 48% had neuropathy [12]. Erasmus et al. [13] in South Africa reported that 20.1% of patients had good glycaemic control which was associated with obesity. Rwegerera [14] in Tanzania reported 24.2% and 32.9% good glycaemic control using HbA_1c_ and fasting/random blood glucose, respectively.

Satisfaction with current antidiabetic treatment was associated with improved glycaemic control among non-insulin-treated type 2 diabetic patients, but gender and participation in a diabetes education program were not [10, 15]. In addition, adherence to antidiabetic drugs significantly increased with an increase in the number of nondiabetic medications [14]. High cost of medication was significantly associated with antidiabetic treatment nonadherence [14]. Good antidiabetic medication adherence was associated with better glycaemic control, but the results were not statistically significant [11]. Other studies elsewhere revealed poor glycaemic control status in most patients and factors such as treatment satisfaction, gender, treatment adherence, DM knowledge, exercise, and obesity were associated with glycaemic control but multiprofessional care and participation in education programs were not [11, 16, 17]. Most studies did not assess reasons for nonadherence, SBGM, and means as influencers of glycaemic control.

In Zambia, diabetes prevalence is estimated to be at <5% but the associated morbidity and mortality at University Teaching Hospital (UTH) were 7.7% and 20.3%, respectively, in 2010 [18, 19]. The reasons for the increasing morbidity and mortality from DM are unclear although poor or sedentary personal lifestyle could be among the factors contributing to this huge burden. However, there is still scarcity of information on glycaemic control patterns and factors that may be associated with it. The poor glycaemic control among diabetic patients compounded with inadequate self-care in Zambia is such an emerging public health concern needing urgent response.

The health care delivery system in Zambia has a pyramid area based structure, with provision of primary health care (PHC) services in lower health facilities such as health posts (HPs) and health centres (HCs) covering a limited geographical area [18]. The PHC services are supported by the first-, second-, and third-level referral hospitals, through an established referral system. Currently, the hospital referral systems are not working as planned. This is largely due to the insufficient capacities at lower levels, including shortages of health workers, erratic supply of essential drugs and medical supplies, and inequities in the distribution of essential physical infrastructure and equipment to offer services that are appropriate to their level, and also due to the limited scope of services offered by facilities at lower levels [18].

In view of the foregoing, Level two hospitals are forced to operate more as district hospitals, as many patients bypass the HPs and HCs due to the observed capacity challenges. Similarly, Level three (tertiary) hospitals are mainly providing first- and second-level hospital services and this situation amounts to inappropriate use of resources, leading to inefficiencies in service delivery [18]. Thus, rightsizing and strengthening the hospital referral systems would result in reductions in congestion at higher level referral facilities and increase in the efficiency and effectiveness of health service delivery [18].

It is believed that prevention and control strategies need to be multithronged in approach and must encompass both individual and group factors despite the evolving theory of change suggesting that individual factors are very critical and probably more than nonindividual factors [20]. There is evidence of efforts focusing on individual factor such as information, education, and communication on SBGM, exercise, diet, and adherence to antidiabetic treatment. Although these have continued to be emphasised as part of the overall management of DM patients, achieving good glycaemic control has continued to be a challenge and has been considered unattainable among some of the diabetic patients [21].

We aimed to assess the current glycaemic control and self-management behaviours that may affect diabetic control among DM patients attending outpatients diabetic clinic at the UTH in Lusaka, Zambia.

## 2. Methods

### 2.1. Design

Data stem from a cross-sectional study, carried out at the UTH diabetic clinic, Lusaka, Zambia. The UTH is the main national referral health centre that treats and reviews patients with various diseases, including DM. The patients attend the diabetic clinic at appointed times as advised by the medical officers for regular monitoring of their disease. All the confirmed diabetic patients on treatment attending outpatients care for at least two years and aged 15 years and above were eligible for the study and newly diagnosed patients that started antidiabetic treatment less than two years were excluded from the study. The patients who agreed to participate in the study provided written informed consent before participating in the study. The study proposal was reviewed and approved by an Independent University of Zambia Biomedical Ethics Committee.

A simple random sampling method was used to accrue eligible consenting patients between September 2013 and December 2013. The sample size and duration of accrual were calculated based on estimated 360 diabetic patients that attend outpatients diabetic clinic and fulfil appointments as well as the proportion of newly referred patients over a four-month period.

Based on Krejcie and Morgan's [22] formula for calculating sample size of a finite population, this gave a calculated sample size of* at least* 186 participants (see sample size calculation below). However, to account for possible exclusions due to refusal to give consent and the need to carry out subgroup analysis, oversampling was ensured to achieve a total of 198 patients who were finally included in the study.

### 2.2. Sample Size Calculation

We have

(1) $$\begin{array}{c} s=\frac{X^{2}NP\left(1-P\right)}{d^{2}\left(N-1\right)}+X^{2}P\left(\;1-P\;\right), \end{array}$$

where *s* is required sample size. *X* ^2^ is the table value of Chi-Square for 1 degree of freedom at the desired confidence level of 0.05 (1.96^2^ = 3.84) (for 95% CI). *N* is the population size. *P* is the population proportion (assumed to be 0.50 since this would provide the maximum sample size). *d* is the degree of accuracy expressed as a proportion (0.05) (±5% accepted error).

Choose

(2) X=1.96,N=360,P=0.50,d=0.05,s=1.962∗360∗0.51−0.50.052360−1+1.962P1−0.5,s=186.

### 2.3. Data Collection

General characteristics: a structured interview schedule was used to capture data on demographic characteristics, self-management behaviours, and laboratory results. The interview schedule was developed based on the World Health Organization (WHO) stepwise survey (STEPS) instrument, version three [23]. The same instruments were used on all the patients to ensure reliability and validity. The data on sociodemographic information were collected using a structured interview schedule, and additional data on clinical factors were extracted from medical records and laboratory results of these patients. Thereafter this data was checked for completeness and entered into the EpiData version 3.0.

The weight and height of the patients were measured using a ZT-160 adult weighing mechanical scale model with a height rod (Wuxi Weigher Factory Co., Ltd., Zhejiang, China) whose values were used to compute the BMI based on the formula developed by Lambert Adolphe Jacques Quételet in 1835 [24]. A scientific calculator FX-82ES (CASIO computer company Ltd., Tokyo, Japan) was used to obtain the actual BMI figure by dividing weight in kilograms with height squared in metres which was also verified by the WHO BMI chart [25].

Laboratory measurement (HbA_1c_): the quantitative determination of HbA_1c_ level in the collected blood from the patients was carried out by the immunoturbidimetry method using the ABX Pentra 400 Automated Clinical Chemistry Analyser (HORIBA ABX SAS, 34184 Montpellier, France), whose technique has been certified by the National Glycohemoglobin Standardisation Program (NGSP) of Australia [26]. The FPG was measured by the enzymatic determination of glucose using the Trinder method using the same analyser [27].

The therapeutic objective of HbA_1c_ has been to obtain values ≤ 48 mmol/mol as recommended by the International Diabetes Federation (IDF) and American College of Endocrinology (ACE) and the target for FPG is ≤ 6 mmol/L [28].

### 2.4. Analyses

Statistical analyses were carried out using IBM SPSS Statistics for Windows version 20.0 (IBM Corp., Armonk, NY, USA). The frequencies and descriptive statistics of the variables were calculated. Pearson's Chi-Squared test, Fisher's exact test, and Student's *t*-test were used to select potential self-management behaviours that may be associated with glycaemic control status. The odds ratio and 95% confidence interval were calculated using multivariate binary logistic regression to identify true potential predictors of glycaemic control while adjusting for confounders. Statistical significance was considered at a *P* value of less than 0.05.

### 2.5. Ethics

This study was approved by the University of Zambia Biomedical Research Ethics Committee on reference number 005-07-13. Written informed consent was obtained from all study participants randomly selected which ensured that all eligible persons were given an equal chance to participate or decline. To ensure confidentiality, the interviews were conducted in preselected private spaces within the health facility and participants' identifiers were kept with the hospital in charge as a standard management protocol but they were ultimately delinked from all research documents except through numerical codes. Venipuncture to obtain blood for testing was considered to pose minimal risk and was acceptable to patients as it was considered a standard practice in the disease management. In similar manner, the patients were not given any direct immediate benefits as they were being interviewed within the hospital environment and at the time that they came for routine referral, consultation, monitoring, or review.

## 3. Results

### 3.1. Patients' Demographic Characteristics

The demographic characteristics of the patients are shown in Table 1. Of all the patients enrolled for the study (*n* = 198), median age was 55 years (IQR 45, 62).

### 3.2. Burden of Diabetes Mellitus

Most (92.9%) of the patients had type 2 DM in contrast to the 7.1% that had type 1 DM. In addition, majority (61.3%) of the patients had poor glycaemic control status and only 38.7% had good glycemic control among those whose data was complete. The mean (SD) FPG of the patients was 9.65 ± 4.96 mmol/L.

### 3.3. Self-Management Behaviours of the Patients

Most (73.7%) of the patients reported not following the treatment regimen as prescribed (adherence) in contrast to the 52 (26.3%) participants that reported adherence to the type of antidiabetic treatment they were on. Only a few (13.1%) patients reported SBGM at home whereas most (86.1%) of them reported none. Amongst the patients who reported SBGM at home, 13 (6.6%) of the patients reported monitoring glucose control at the public health facility, 2.5% own glucometer, and 4.0% reported glucose monitoring at the private health facility. The majority (59.6%) of the patients were not involved in any type of regular physical exercise.

### 3.4. Glycaemic Control Status by Characteristics/Self-Management Behaviours

The glycaemic control status of the patients according to the characteristics and self-management behaviours is shown in Table 2. In bivariate analysis, there was an association between glycaemic control status and adherence to treatment and FPG. However, there was no association between age, sex, education, BMI, SBGM, means of SBGM, exercise, and glycaemic control status.

### 3.5. Predictors of Glycaemic Control Status

The multivariate binary logistic regression model was tested for multicollinearity, Hosmer and Lemeshow test of model fitness for data, and omnibus test of model coefficients and classification accuracy. The dependent variable was glycaemic control status: Good (1) and Poor (0). The results of the multivariate binary logistic regression analysis to predict whether the 9 variable factors, age, sex, education level, BMI, adherence to treatment, SBGM, SBGM means, and exercise, predicted glycaemic control status showed that only adherence to treatment and FPG predicted glycaemic control status of the patients (Table 3). Thus, the patients who do not adhere to antidiabetic treatment and those with mean (SD) FPG, 10.26 ± 5.17 mmol/L, are 68% and 7% less likely to achieve good glycaemic control status (Table 3).

## 4. Discussion

It is well established that nonadherence rates for chronic disease regimens and for lifestyle changes are generally approximately 50%, and patients with diabetes are particularly prone to regimen adherence problems especially when on multiple treatment regimens including medications, lifestyle, diet, and exercise [30–33]. Consequently, successful management of DM is challenging, yet patients with good self-care behaviours can achieve excellent glycaemic control and avoid frequent diabetic complications.

However, many patients are devoid of optimal self-care behaviours and continue to suffer from complications of the disease. Regular SBGM empowers patients to play a role in the management of diabetes, simultaneously improving their metabolic parameters. Glucometers are frequently used to assess the FPG and the results can prompt and help the patient to adjust the diet (especially carbohydrate intake), exercise, and improve adherence to or modify medication dosage. The consistent use and correct response to the glycaemic results have been shown to improve glycaemic control in type 2 diabetes, consequently preventing or delaying further complications of diabetes [34].

In this study we found evidence of poor glycaemic control which was significantly associated with poor adherence to medication use among diabetic patients that regularly attend medical review at the diabetic clinic. However, poor glycaemic control was not strongly associated with age, sex, education level, SBGM and means of SBGM, exercise, and BMI, associations that have been observed elsewhere [35–37]. While the present study showed higher HbA_1c_ values in patients aged over 54 years as well as those that attained at least secondary education, the results were not statistically significant. However, adherence to antidiabetic treatment and FPG were statistically significantly associated with higher HbA_1c_ values.

These findings are not surprising given that as a developing nation Zambia lacks the resources and capacity to manage this disease which could be associated with adherence [38].

While developed nations have processes, strategies, and infrastructures imbedded in their health care programs, including electronic continuous monitoring technology, that allow medical management of both early and late stage disease, developing nations have inadequate capability to manage and reverse the increasing morbidity and mortality [39]. This association with adherence to antidiabetic treatment and FPG observed in this study is a common problem among individuals with diabetes [40–43]. It is therefore necessary to determine the effective behavioural interventions that can improve adherence in the Zambian diabetic patients. Consequently, this study has persuaded us to consider a focus for a changed interventional approach targeting selected factors including patient centered approaches such as understanding patient insight of the disease, and collaborative and clear communication between health care professionals and patients could impact positively on glycaemic control in these patients.

Firstly, we are aware that this was a cross-sectional study and therefore it is difficult to establish a “causal” relation between HbA_1c_ and the self-management behaviours.

Secondly, we also acknowledge that this was a small and highly selected sample from a frame of hospital attendees at a referral tertiary hospital. This limitation was augmented by the fact that the cost of purchasing laboratory materials and supplies for the study provided additional sample size limitations.

Thirdly and in addition, we also observe that there could be additional biological individual variations which could potentially bias our measurements.

Fourthly and lastly though not the least, we also report that there was incomplete data on medical records of the diabetic outpatients at the clinic, making it difficult to follow the morbidity and mortality patterns and thus reducing further the possible sample which would have improved the study power and understanding of possible determinants.

Notwithstanding the possible presence of such selection and measurement biases, we do not think these could have been important in explaining the findings as their effects are assumed to have been non-differentially distributed given the random selection of subjects that was used.

In Zambia the mortality of patients with type 2 DM is likely to continue to increase as the Zambian economy improves a factor associated with increase in western life style including diet and sedentary way of life [44]. A needs' assessment for the Noncommunicable Disease program carried out by the MoH identified deficiencies in diabetic control as having inadequacies in terms of drugs and laboratory reagents, diagnostic facilities, expertise, and community awareness for DM [18]. It may thus be not surprising to find presence of individual factors associated with poor glycaemic control status where we further argue that these are also associated with not only system supply factors but also social factors, predominantly operating at an individual level. Consequently, there is need to define DM disease burden and epidemiology with focus on determinants and thus determine potential strategies that could address these inadequacies and improve the disease outcome.

The majority of diabetic outpatients in this study had poor glycaemic control status and this could be due to a variety of factors including lack of resources and ability to buy diabetic chips and strips that allow for more frequent checks of FPG levels and/or ability to have adequate resources to store, for example, the insulin in a refrigerator which is frequently not available to many Zambians [45]. The reasons for this were not the focus of this paper and so we may only speculate regarding possible reasons for these differential glycaemic control status associations. We, however, disagree with what other evidence suggest that this can also probably be because of poor diet and exercise habits and other multiple barriers [46]. However, good glycaemic control was reported in Japan and Germany also and it has been argued that perhaps this is because of higher literacy levels in these countries resulting in improved knowledge translation about DM [47, 48].

It was interesting to note that there was a statistically significant association between adherence to antidiabetic treatment and glycaemic control status of the diabetic outpatients in this study.

This is important given that the effectiveness of drug treatment depends primarily on the efficacy of the prescribed treatment and adherence of the patient to the treatment [49]. These findings have been supported by studies elsewhere which have shown that adherence to antidiabetic treatment among diabetic patients is poor and the possible reasons have been outlined. One of the reasons is simply failure to understand and consequently failure to comply with the prescribed clinical regimen, thereby resulting in very poor outcomes [40].

In the present study, this was illustrated clearly where we observed that the diabetic outpatients who did not adhere to DM treatment had 68% decrease in the likelihood of achieving good glycaemic control status compared to those who adhered. In similar manner, in the study by Ahmad et al. [35] in Malaysia, 53.0% of the patients were nonadherent to DM treatment and the main factor which was associated with that nonadherence was age. In another study by Curkendall et al. [50] in the US, it revealed that only 45% of the patients were adherent to DM treatment. The adherence was high in the males, older patients, or patients residing in specific geographical area. The factors related to poor adherence were comorbidity, overall health level, number of drugs, and complexity of the drug regimen [51].

There is thus a critical need to understand adherence related factors so as to design prevention and control strategies that will be operable but accounting for contextual differentials even across low income countries in general. If adherence is improved this could positively improve lives of these clients. In fact, some studies have shown that an increase in adherence by as little as 10% can decrease the levels of HbA_1c_ significantly [35, 51]. Such increase is possible if behavioural linked factors such as education attainment, which has already been shown to improve glycaemic control status, are targeted [51, 52], However, tackling nonadherence is not a simple matter, as it is multifactorial and might include cost adjustments, health belief transformations, dosing frequency repackaging, and assessment of the presence of potential confounders associated with personality disorders and patient-provider relationship [40].

There is need to explore the effective behavioural interventions that can improve adherence to antidiabetic treatment among the diabetic patients in Zambia. Changing interventional approaches targeting selected factors including patient centered approaches such as understanding patient insight of the disease and collaborative and clear communication between health care professionals and patients could impact positively on glycaemic control in diabetic patients. Thus, the role of diabetic patients in the management of their diabetes remains paramount.

If this matter is critically managed, it is possible that the outcome of treatment would be much more satisfactory among diabetic outpatients and this could possibly delay the development of the complications of DM and improve the quality and length of lives for the affected individuals. There is thus need to institute prevention and control mechanism that are cost-effective, acceptable, and appropriate. Thus, combined screening with FPG and HbA_1c_ used in this study may identify patients at very high risk for diabetes when FPG and HbA_1c_ are considered together.

## 5. Conclusion

We conclude that, among diabetic patients, poor glycaemic control remains a challenge and this may to a greater extent be associated with “adherence to antidiabetic treatment” related factors. This may suggest limitations in past prevention and control efforts for DM at individual level as well as at care level where monitoring dynamics are limited as these patients are largely outpatients except when they are admitted to hospital for one reason or another. However, finding that FPG predicted the glycaemic control opens potential opportunities to routinely examine and identify most at risk groups, which in turn and further opens possibilities to study the associated dynamics linked to care and support. This could in turn therefore inform interventional policies and control strategies and thus improve the overall care and support of such patients. In addition, this may also be important to identify prediabetic states.

Furthermore, and given that the self-management behaviours of diabetic patients play an important role in the management of DM, there is thus need to target improvement of the efficacy of individual strategies in all prevention and control strategies for these patients.

We further argue that if this is done properly, it may consequently reduce diabetic complications and thus improve the lives of these people. The health care providers also are critical stakeholders and need to foster and place greater emphasis on counselling and improving adherence, notwithstanding the context specific differences.

## Figures and Tables

**Table 1:** Demographic characteristics of the patients (*n* = 198).

Variable	Frequency	Percent
Age		
15–34 years	22	11.1
35–54 years	77	38.9
55 years and above	99	50.0
Total	**198**	**100**
Sex		
Male	79	39.9
Female	119	60.1
Total	**198**	**100**
Education level		
Never/primary	74	37.4
Secondary	92	46.5
College/university	32	16.2
Total	**198**	**100**
^*∗*^Body mass index (kg/m^2^) (*n* = 190)		
Underweight (≤18.4 kg/m^2^)	6	3
Normal (18.5–24.9 kg/m^2^)	60	30.3
Overweight (25–29.9 kg/m^2^)	70	35.4
Obese (30 or greater kg/m^2^)	54	27.3
Total	**190**	**100**

^*∗*^According to the WHO classification of obesity [29].

**Table 2:** Glycaemic control status by characteristics/self-management behaviours of the participants at the University Teaching Hospital.

Characteristic/self-management behavior	Glycaemic control status		*P* value^*∗*^
	Good (*n* = 75, HbA_1c_ ≤ 48 mmol/mol)	Poor (*n* = 119, HbA_1c_ ≥ 49 mmol/mol)	
	No (%)	No (%)	
Age^a^			
15–34 years	4 (19.0)	17 (81.0)	
35–54 years	29 (38.2)	47 (61.8)	0.117
55 years and above	42 (43.3)	55 (56.7)	
Sex^a^			
Male	29 (38.2)	47 (61.8)	
Female	46 (39.0)	72 (61.0)	0.908
Education level^a^			
Never/primary	27 (36.5)	47 (63.5)	
Secondary	33 (37.5)	55 (62.5)	0.575
College/university	15 (46.9)	17 (53.1)	
Adherence^a^ (*N* = 192)			
No	44 (30.8)	99 (69.2)	
Yes	31 (60.8)	20 (39.2)	**0.000**
SBGM^a^			
No	63 (37.5)	105 (62.5)	
Yes	12 (46.2)	14 (53.8)	0.399
SBGM means^b^			
Owning glucometer	3 (60.0)	2 (40.0)	
Public health facility	6 (46.2)	7 (53.8)	0.686
Private health facility	3 (37.5)	5 (62.5)	
Not applicable	63 (37.5)	105 (62.5)	
Exercise^a^			
No	47 (41.2)	67 (58.8)	
Yes	28 (35.0)	52 (65.0)	0.381
BMI (kg/m^2^)^b^ (*N* = 186)			
Underweight (≤18.4)	1 (16.7)	5 (83.3)	
Normal (18.5–24.9)	17 (30.4)	39 (69.6)	
Overweight (25–29.9)	31 (44.3)	39 (55.7)	0.306
Obese (≥30)	22 (40.7)	32 (59.3)	
FPG (mmol/L; mean, SD)^c^	8.47 (3.88)	10.26 (5.17)	**0.011**

^a^Pearson's Chi-Squared test, ^b^Fisher's exact test, and ^c^Student's *t*-test. ^*∗*^Significant *P* value at *P* < 0.05, SBGM: self-blood glucose monitoring, BMI: body mass index, FPG: fasting plasma glucose, and SD: standard deviation.

**Table 3:** Multivariate binary logistic regression model-determining predictors of glycaemic control status.

Predictor variable	Glycaemic control status		AOR (95% CI)	*P* value^*∗*^
	Good (*n* = 75, HbA_1c_ ≤ 48 mmol/mol)	Poor (*n* = 119, HbA_1c_ ≥ 49 mmol/mol)		
	No (%)	No (%)		
Adherence				
No	44 (30.8)	99 (69.2)	0.32 (0.16–0.63)	**0.001**
Yes	31 (60.8)	20 (39.2)	Ref (1.00)	
Current FPG (mmol/L; mean, SD)	8.47 (3.88)	10.26 (5.17)	0.93 (0.86–1.00)	**0.046**

HbA_1c_: glycated haemoglobin, FPG: fasting plasma glucose, SD: standard deviation, Ref: reference category, mmol/L: millimoles per litre, and mmol/mol: millimoles per mole. ^*∗*^Significant *P* value at *P* < 0.05. AOR: adjusted odds ratio; CI: confidence interval.
